# Demographic trends and disparities among NIH-funded medical school faculty in the US, 1970–2022

**DOI:** 10.1371/journal.pone.0337610

**Published:** 2025-12-01

**Authors:** Alyssa Browne

**Affiliations:** Association of American Medical Colleges (AAMC), Washington, DC, United States of America; Iowa State University, UNITED STATES OF AMERICA

## Abstract

**Background:**

Although gender and racial/ethnic trends and disparities are well documented among recipients of National Institutes of Health (NIH) research grant funding, little is known about the demographic trends and disparities within a critical subpopulation: NIH-funded medical school faculty.

**Methods:**

This paper analyzes a rich dataset of full-time medical school faculty and their NIH grants from 1970 to 2022 and examines temporal trends in the demographic distribution and relative representation (representation index) of NIH-funded medical school faculty by gender, race/ethnicity, and their intersections.

**Results:**

This paper reports that, while the gender gap in NIH funding has narrowed, a gender gap remains, particularly among highly funded faculty. Further, trends disaggregated by both gender and race/ethnicity demonstrate the particularly low relative representation of Black and Hispanic NIH-funded faculty, specifically Black and Hispanic women, that persists over time. Notably, although Asian men and women are represented at similar rates among medical school faculty overall, a gender gap in favor of Asian men has become more pronounced over time among NIH-funded Asian faculty.

**Conclusions:**

Although there are efforts to recognize a broader set of metrics for a successful research career, research grant funding remains key for establishing research independence, tenure, and promotion. Thus, our findings of ongoing disparities underscore the urgent need to identify effective strategies to advance gender and racial/ethnic equity within NIH-funded medical school faculty.

## 1. Introduction

In recent years, a growing body of research has demonstrated that biomedical science benefits from diversity [1–5]. Yet, addressing gender and racial/ethnic disparities to build a more diverse biomedical research workforce remains a well-documented challenge in the United States (U.S.) [4,6], as reflected in persistent gender and racial/ethnic disparities in the likelihood of NIH applicants receiving grant funding [7–9] and in the probability of holding multiple NIH research project grants (RPGs) concurrently – sometimes referred to as being a “super investigator” [10]. In 2022, only 38% of NIH-funded research grant investigators were women [11], and only 3.2% of NIH-funded principal investigators (PIs) were Black or African American, despite 14.4% of the U.S. population self-identifying as Black that same year [12]. Similar patterns of underrepresentation are observed for Hispanic/Latinx, American Indian and Alaskan Native, and Pacific Islander NIH-funded PIs [13].

This paper addresses two critical gaps in our knowledge about the NIH-funded biomedical workforce. First, we know little about what NIH funding trends look like disaggregated by both gender and race/ethnicity. Intersectionality recognizes that race/ethnicity, gender, and other socially defined categories operate together to shape social inequalities [14], meaning that we might expect to find crucial complexity in the data when we go beyond considering only one dimension of inequality. For example, a recent analysis that disaggregated NIH funding by both gender and race/ethnicity found that Black women experienced the largest disparity of all groups [10], and a study focused on biomedical authorship likewise found racial/ethnic variation in the disparities experienced by women [15]. Research findings like these motivate this paper, which examines NIH funding disparities disaggregated by both gender and race/ethnicity.

Second, this paper focuses on a critical sub-population of the NIH-funded workforce, about whom we know little: NIH-funded faculty at medical schools. Medical schools – and medical school faculty – are key contributors to the biomedical research workforce, receiving approximately 60% of NIH funding [16]. Often bridging biomedical discovery and clinical care [17], they are uniquely positioned to drive innovation. Additionally, research undertaken by teams of diverse medical school faculty has contributed to improved clinical outcomes for racially and ethnically diverse populations [18,19]. It is thus imperative to understand trends in the demographic composition of NIH-funded medical school faculty. While we have some recent data on NIH-funded medical school faculty, they are limited to faculty with an R01, a highly-competitive type of research and development grant awarded to support a discrete research project [20].

This paper pursues three aims: First, to describe the trends in gender, race/ethnicity, and joint gender-race/ethnicity distribution of NIH-funded full-time faculty at U.S. accredited medical schools from 1970 to 2022. For this analysis, the author defines NIH-funded faculty as faculty with at least one NIH research project grant (RPG), grants that support specific biomedical research projects performed by principal investigators [21]. Second, to compare trends in representation among NIH-funded U.S. medical school faculty to trends in representation within the medical school faculty overall. Third, to understand trends at a more granular level by comparing trends in representation for NIH-funded faculty with less than three RPGs to trends among NIH-funded faculty with three or more RPGs, who are sometimes called “super investigators” [10].

## 2. Materials and methods

### 2.1 Study design and sample

This paper analyzes a unique dataset that merges AAMC Faculty Roster data with NIH grants data, for nearly all full-time medical school faculty in the U.S. from fiscal year 1970 through fiscal year 2022. The merging of the data was done under a contract between the AAMC and NIH (75N94019C00007), with the NIH conducting the merge and with the AAMC contributing to the checking and refining of the merge methodology. This dataset includes 486,576 unique individuals who were full-time medical school faculty within the observed period, 60,242 (12.4%) of whom had NIH RPG funding at some point within their faculty career. The dataset includes identifiable person-level data, which is only shared or published in a de-identified form.

This dataset has several key strengths. First, rather than representing a sample of faculty, the AAMC Faculty Roster represents nearly all full-time medical school faculty in the U.S. during this 53-year period, collected on a rolling basis. Second, this faculty dataset is merged with complete data on every full-time faculty member’s NIH-funded RPGs during the same period. Third, self-reported data on key demographic characteristics and a large population allow for intersectional analysis by gender and race/ethnicity. The large dataset also permits comparisons of NIH-funded faculty according to the number of NIH grants simultaneously awarded during any given year. It is important to specify that this dataset does not include data on applications, so it is not possible to calculate success rates, nor the probability of applications being funded. Rather, this paper examines trends in the representation of specific demographic groups within medical school faculty who were awarded research funds from NIH.

### 2.2 Measures

The two self-reported demographic measures the author analyzed, gender and race/ethnicity, are from the AAMC Faculty Roster data and come from Faculty Roster representatives at each medical school. If a medical school is not able to provide demographic data from its administrative databases, the AAMC can often backfill the demographic data from other AAMC applications where individuals report their demographic information. For this analysis, the gender variable is a binary measure of men and women, the categories reported by institutions. The racial and ethnic measure is a combined measure that includes faculty who identified as non-Hispanic Asian only (“Asian”), non-Hispanic Black or African American only (“Black”), non-Hispanic White only (“White”), Hispanic or Latino only (“Hispanic”), multiple races/ethnicities (“Multiracial/ethnic”), and other races/ethnicities (“Other”). American Indian/Alaska Native and Native Hawaiian and other Pacific Islander faculty, representing 1,304 faculty (0.27%), were combined with “other” race faculty due to small numbers. The author excluded faculty for whom race/ethnicity and/or gender was unknown (N = 18,289, 3.8%).

This analysis uses PI-level NIH grants data, which includes data on every active NIH grant on which a full-time faculty member was a sole or co-PI during a given fiscal year. For the analysis of group-level trends, the key measure of interest was an indicator variable denoting whether a grant was an RPG. From this, the author generated a fiscal-year specific indicator variable for each faculty member that specifies if a faculty member had at least one NIH RPG during a given fiscal year (yes = 1; no = 0). This measure allowed the identification of NIH-funded faculty. To examine trends disaggregated by super investigator status, the author counted all active RPGs on which a faculty member was a PI in a given fiscal year and generated a variable denoting whether a PI was a super investigator (0 = no, < 3 RPGs; 1 = yes, 3 + RPGs).

### 2.3 Statistical analysis

All analysis was completed in Stata Version 14.2. The author analyzed temporal trends in the gender and racial/ethnic distribution of all full-time faculty and faculty with NIH funding at medical schools. The author also dichotomized NIH-funded faculty according to the number of concurrent RPGs (<3 and 3+) held. To compare the demographic distribution of NIH-funded faculty to all full-time medical school faculty over the period studied, the author calculated demographic group-specific representation index (RI) values. The RI (sometimes called the “representation quotient” [22,23]) is a measure of disproportionate representation [24] that, as currently applied, directly compares the representation of each gender, race/ethnicity, and gender-race/ethnicity group within NIH-funded faculty to that same demographic group’s representation among all medical school faculty using the following formula:


$$\text{RI}\;=\frac{\text{Percentage}\;\text{of}\;\text{demographic}\;\text{group}\;\text{among}\;\text{NIH}-\text{funded}\;\text{medical}\;\text{school}\;\text{faculty}}{\text{Percentage}\;\text{of}\;\text{demographic}\;\text{group}\;\text{among}\;\text{medical}\;\text{school}\;\text{faculty}}$$

An RI of less than one indicates that that demographic group is underrepresented among NIH-funded faculty relative to that same demographic group’s representation among medical school faculty. A value of one indicates equivalent representation and a value over one indicates relative overrepresentation among NIH-funded faculty. Further, the RI makes the extent of under- and over-representation interpretable [23]. An RI of 0.8 means that the representation of that demographic group among NIH-funded faculty is 80% of that same group’s representation among all full-time medical school faculty, while an RI of 0.4 – indicating representation that is 40% relative to that group’s representation among medical school faculty – implies starker underrepresentation. The author examined temporal trends in the distribution by demographic characteristics of all NIH-funded faculty and NIH-funded faculty disaggregated by the number of active grants (<3, 3+).

To evaluate whether trends over time were statistically significant for each demographic group, the author regressed each demographic indicator (e.g., race/ethnicity composition; RI) on fiscal year, including interaction terms between fiscal year and the relevant demographic indicator. To assess differences in trends between groups, marginal effects and confidence intervals were calculated post-estimation. Because the data reflect the full population of NIH-funded medical school faculty rather than a sample, values at individual time points were not subjected to the same statistical testing, as they represent true observed values rather than estimates.

### 2.4 Ethical considerations

This study was approved by the AAMC Institutional Review Board (IRB) and the author accessed the dataset on January 15, 2024.

## 3. Results

**Fig 1:**
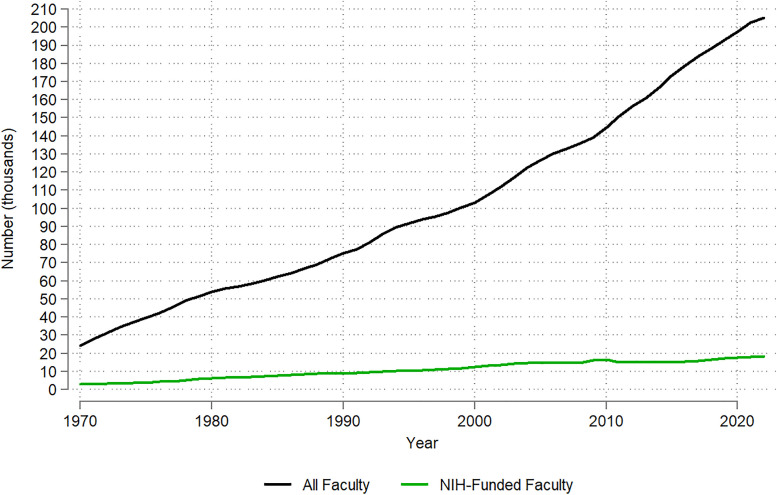
Temporal trends in the number of full-time U.S. medical school faculty (total and with NIH funding): 1970-2022.

Over the 53-year period studied, the overall growth among medical school faculty outpaced growth among faculty with NIH funding (Fig 1), possibly reflecting a shift among medical school faculty away from research faculty tracks and toward clinical and instructional faculty tracks [25]. In 1970, there were 24,311 full-time medical school faculty, 2,881 (11.9%) of whom were NIH-funded. By 2022, there were 205,379 full-time medical school faculty, 18,320 (8.9%) of whom were NIH-funded. Overall, there was a 745% increase in the number of faculty between 1970 and 2022 and a 536% increase in the number of NIH-funded faculty in that same period.

The following section presents descriptive trends in the demographics of NIH-funded medical school faculty between 1970 and 2022. It includes the distributions of faculty with NIH funding by gender, race/ethnicity, and their intersections, as well as trends in NIH-funded faculty’s relative representation compared to the broader medical school faculty, measured using the Representation Index (RI). Trends are shown for all NIH-funded faculty and stratified by funding level: those with fewer than three RPGs and those with three or more (“super investigators”). For detailed annual frequency and demographic breakdowns, see S1-S4 Tables, which provide data for [1] all medical school faculty, [2] all NIH-funded faculty, [3] NIH-funded faculty with <3 RPGs, and [4] NIH-funded faculty with 3 + RPGs.

**Fig 2:**
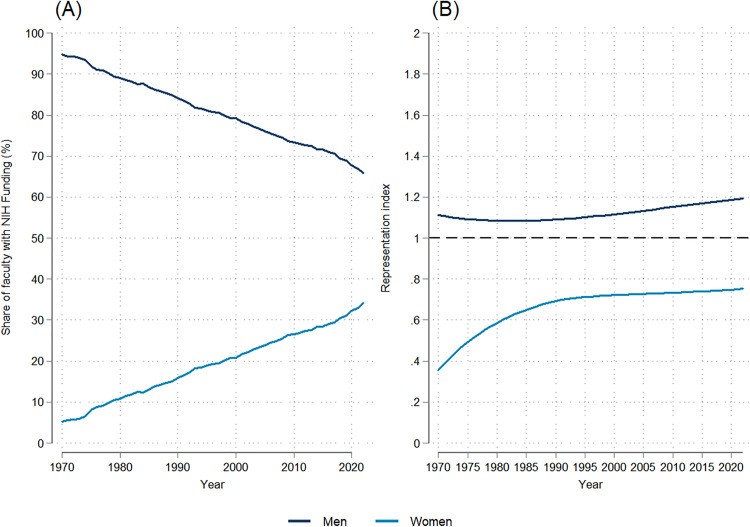
Temporal trends in the gender distribution and representation index of NIH-funded full-time medical school faculty: 1970-2022. (A) Trends in the gender distribution of NIH-funded faculty. (B) Trends in the representation index by gender.

Gender trends show that, among faculty who received NIH funding, there was a steep decline between 1970 and 2022 in the proportion who were men, from 94.7% in 1970 to 65.8% in 2022 (Fig 2A), a trend that was statistically significant (dy/dx = −0.532, p = 0.000; S5 Table). However, since men remained more highly represented among NIH-funded faculty relative to their representation among medical school faculty overall, their RI increased slightly between 1970 and 2022 (dy/dx = 0.002, p = 0.000; S5 Table), from 1.1 to 1.2 (Fig 2B).

As men’s share among NIH-funded faculty declined over the period studied, there was a corresponding statistically significant upward trend (dy/dx = 0.53, p = 0.000; S5 Table) in the proportion of NIH-funded faculty who were women, with women comprising approximately one-third (34.2%) of faculty who received NIH funding by 2022. Between 1970 and 2022, the relative representation of women with NIH funding increased at a steeper rate (dy/dx = 0.005, p = 0.000; S5 Table) than for men, from an RI of 0.36 to 0.75 (Fig 2B). This means, however, that in 2022 women’s representation among NIH-funded faculty remained only 75% that of their representation among medical school faculty overall, while men remained overrepresented.

**Fig 3:**
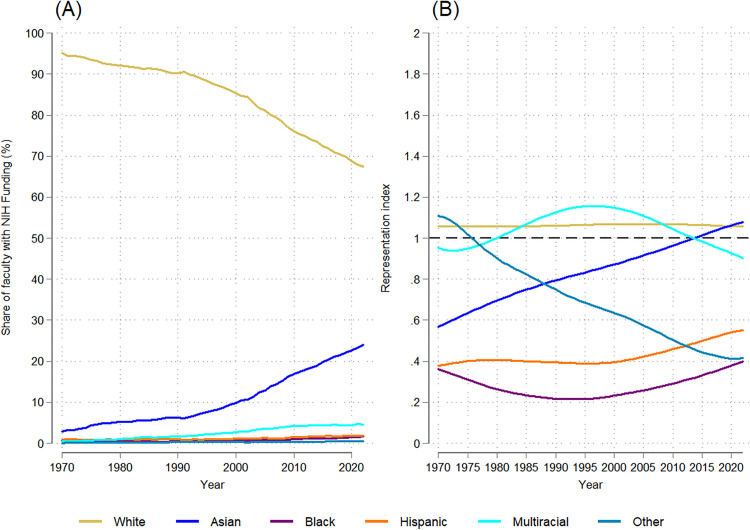
Temporal trends in the racial/ethnic distribution and representation index of NIH-funded full-time medical school faculty: 1970-2022. (A) Trends in the racial/ethnic distribution of NIH-funded faculty. (B) Trends in the representation index by race/ethnicity.

Demographic trends by race/ethnicity mirrored those by gender: the group initially disproportionately represented (in this case, white faculty) saw steady declines over the 53-year period. In 1970, white faculty comprised nearly all (95%) NIH-funded medical school faculty; by 2022, this had dropped to 67% (Fig 3A), a statistically significant downward trend (dy/dx = −0.537, p = 0.000; S5 Table). This decline mirrored broader shifts in the medical school faculty population, so the relative representation of white faculty remained stable, with no significant trend, and RI values that remained between 1 and 1.1 (Fig 3B).

The proportion of NIH-funded faculty who were Asian increased 21 percentage points, from 2.9% to 23.9% (dy/dx = 0.401, p = 0.000; S5 Table). While Asian faculty were underrepresented relative to their share of medical school faculty in 1970 (RI = 0.6), they surpassed equal representation by 2022 (RI = 1.1), reflecting a statistically significant upward trend in relative representation (dy/dx = 0.009, p = 0.000; S5 Table).

In contrast, Hispanic, Black, multiracial/ethnic, and other race faculty each accounted for 5% or less of NIH-funded faculty throughout the period studied (Fig 3A; See S1 Fig for closer detail). Multiracial/ethnic faculty experienced a statistically significant increase in share (dy/dx = 0.092, p = 0.000; S5 Table), although their relative representation fluctuated without a clear or significant trend, between 0.8 and 1.3. Black, Hispanic, and other race faculty remained consistently underrepresented. For other race faculty, the RI dropped below 1 by 1981 and declined over time (dy/dx = −0.013, p = 0.000; S5 Table), while the RI for Hispanic faculty never exceeded 0.6, despite a slight positive trend (dy/dx = 0.002, p = 0.003; S5 Table). Notably, Black faculty had the lowest relative representation of all groups, with an RI that never exceeded 0.4–40% of their level of representation within medical school faculty broadly – and no significant trend over time. That Black and Hispanic faculty remained so underrepresented among NIH-funded faculty after 53 years is particularly notable given their already limited presence among medical school faculty.

**Fig 4:**
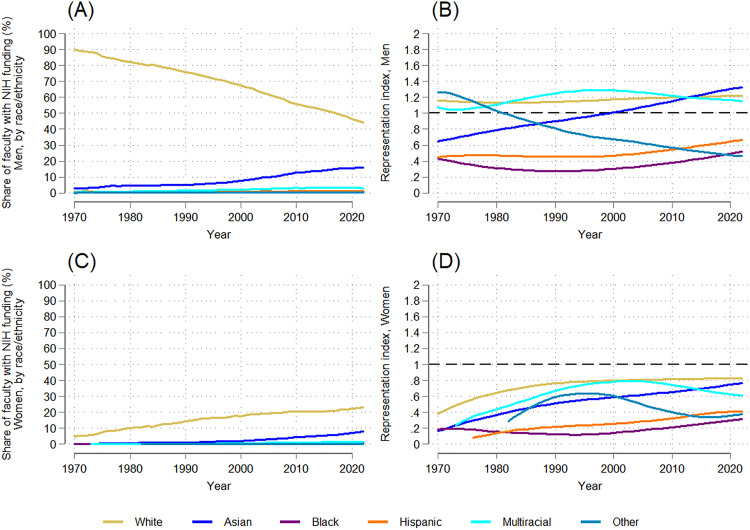
Temporal trends in the gender-racial/ethnic distribution and representation index of NIH-funded full-time medical school faculty: 1970-2022. (A) Trends in the distribution of NIH-funded faculty (men, by race/ethnicity). (B) Trends in the representation index by race/ethnicity (men). (C) Trends in the distribution of NIH-funded faculty (women, by race/ethnicity). (D) Trends in the representation index by race/ethnicity (women).

Disaggregating the data by both gender and race/ethnicity provides a more complete picture of how NIH-funded medical school faculty have evolved over time. In 1970, white men accounted for a staggering 90% of NIH-funded medical school faculty (Fig 4A); by 2022, this dropped to 44% (Fig 4A), a statistically significant negative trend (dy/dx = −0.876, p = 0.000; S5 Table). Over the same period, Asian men’s share rose from 2.7% of NIH-funded faculty to 16.1% (dy/dx = 0.267, p = 0.000; S5 Table), while multiracial/ethnic men remained a small share (< 3%) but trended upward (dy/dx = 0.061, p = 0.000; S5 Table). In contrast, Black, Hispanic, and other race men consistently represented 3% or less of NIH-funded faculty (Fig 4A; See S2 Fig for closer detail), and showed no significant trends (S5 Table).

White men remained consistently overrepresented among NIH-funded faculty relative to their representation among medical school faculty overall (RR > 1; slope = 0.002, p = 0.036) (Fig 4B; S5 Table). Asian men surpassed proportional representation by 2022, with an RI of 1.3 (slope = 0.013, p = 0.000; S5 Table), while multiracial/ethnic men’s RI fluctuated between 0.9 and 1.5 and increased slightly overall (dy/dx = 0.003, p = 0.004; S5 Table). Other race men’s RI declined to 0.5 by 2022 (dy/dx = −0.015, p = 0.000). Finally, Black and Hispanic men remained underrepresented relative to their share of the medical school faculty throughout the period studied, with RIs never exceeding 0.5 or 0.7, respectively, though both groups of men saw slightly positive trends (Black men: slope = 0.003 p = 0.008; Hispanic men: slope = 0.003, p = 0.001; S5 Table). Racial/ethnic variation in RI trends among men are substantial: while men overall are overrepresented among NIH-funded faculty, this is not universal across racial/ethnic groups. Instead, persistent disparities see Black, Hispanic, and other race men remaining markedly underrepresented.

White women experienced notable gains in their share of NIH-funded faculty – from 5% to 23% (Fig 4C), a statistically significant positive trend (dy/dx = 0.340, p = 0.000; S5 Table) – while Asian women’s share increased from 0.2% to 8% (dy/dx = 0.134, p = 0.000; S5 Table). Black, Hispanic, multiracial/ethnic, and other race women each remained at 3% or less of all NIH-funded faculty across all years. Multiracial/ethnic women saw a trend upward (dy/dx = 0.032, p = 0.000; S5 Table); trends observed for Black and Hispanic women were significantly shallower and not significant.

As a result, every racial/ethnic group of women remained underrepresented (RI < 1) relative to their share of medical school faculty (Fig 4D), in contrast to the relative representation of white, Asian, and multiracial/ethnic men. White women’s RI increased from 0.4 to 0.9 (dy/dx = 0.006, p = 0.000; S5 Table), while Asian women’s RI increased more steeply from 0.2 to 0.8 (dy/dx = 0.010, p = 0.000; S5 Table), a statistically significant difference in RI trends relative to white women. Multiracial/ethnic women’s RI fluctuated, but increased overall (dy/dx = 0.007, p = 0.000; S5 Table), while other race women’s RI ultimately decreased (dy/dx = −0.007, p = 0.000; S5 Table). Black and Hispanic women remained the most underrepresented, with RIs that never exceeded 0.4 despite slight upward trends (Black women: slope = 0.002, p = 0.020; Hispanic women: slope = 0.006, p = 0.000; S5 Table). Notably, the RI values of Black and Hispanic women fell below the RIs of men of their same race/ethnicity, meaning Black and Hispanic women experienced the starkest disparities in NIH funding across all gender-race/ethnicity groups studied.

**Fig 5:**
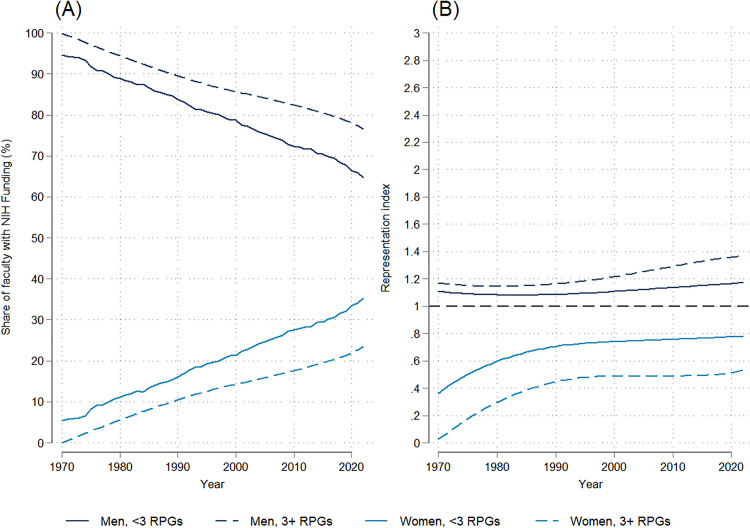
Temporal trends in the gender distribution and representation index of NIH-funded full-time medical school faculty with <3 RPGs and 3 + RPGs: 1970-2022. (A) Trends in the gender distribution of NIH-funded faculty with <3 RPGs and 3 + RPGS. (B) Trends in the representation index by gender, faculty with <3 RPGs and 3 + RPGs.

**Fig 6:**
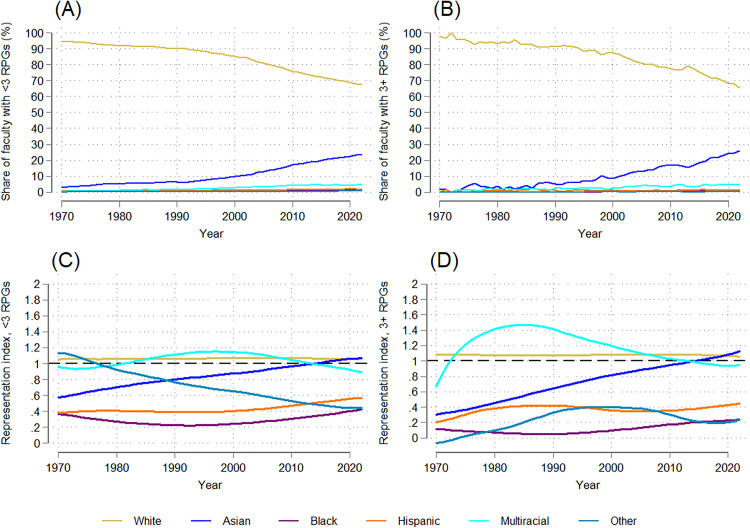
Temporal trends in the racial/ethnic distribution and representation index of NIH-funded full-time medical school faculty with <3 RPGs and 3 + RPGs: 1970-2022. (A) Trends in the racial/ethnic distribution of NIH-funded faculty with <3 RPGs. (B) Trends in the racial/ethnic distribution of NIH-funded faculty with 3 + RPGs. (C) Trends in the representation index by race/ethnicity of NIH-funded faculty with <3 RPGs. (D) Trends in the representation index by race/ethnicity of NIH-funded faculty with 3 + RPGs.

**Fig 7:**
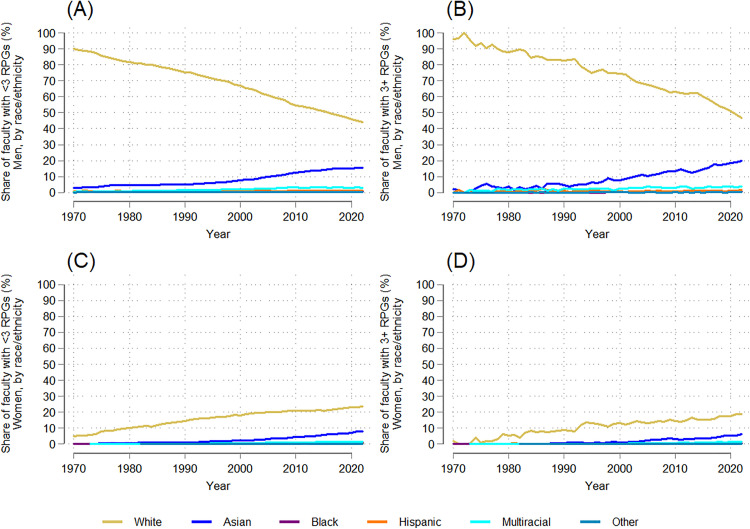
Temporal trends in the gender-racial/ethnic distribution of NIH-funded full-time medical school faculty with <3 RPGs and 3 + RPGs: 1970-2022. (A) Trends in the distribution of NIH-funded faculty with <3 RPGs (men, by race/ethnicity). (B) Trends in the distribution of NIH-funded faculty with 3 + RPGs (men, by race/ethnicity). (C) Trends in the distribution of NIH-funded faculty with <3 RPGs (women, by race/ethnicity). (D) Trends in the distribution of NIH-funded faculty with 3 + RPGs (women, by race/ethnicity).

**Fig 8:**
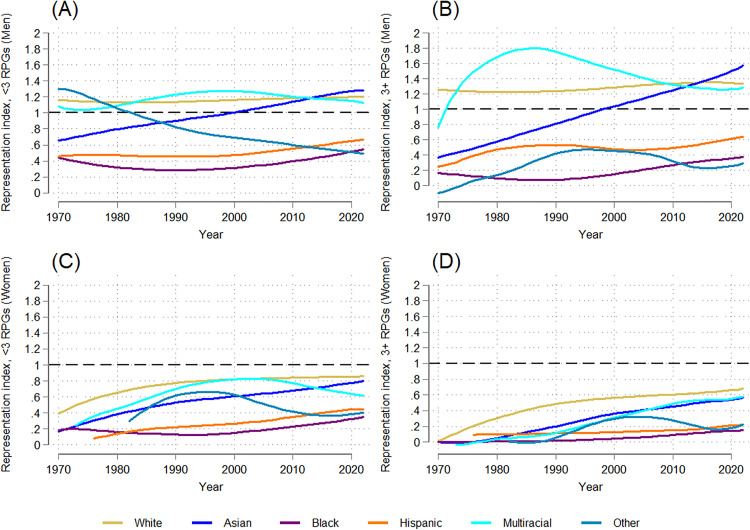
Temporal trends in the gender-racial/ethnic representation index of NIH-funded full-time medical school faculty with <3 RPGs and 3 + RPGs: 1970-2022. (A) Trends in the representation index by race/ethnicity of NIH-funded faculty with <3 RPGs (men). (B) Trends in the representation index by race/ethnicity of NIH-funded faculty with 3 + RPGs (men). (C) Trends in the representation index by race/ethnicity of NIH-funded faculty with <3 RPGs (women). (D) Trends in the representation index by race/ethnicity of NIH-funded faculty with 3 + RPGs (women).

To assess whether disparities were narrower among faculty with fewer RPGs than among NIH super investigators, the author stratified the data by the number of concurrent RPGs faculty held at one time (Figs 5, 6, 7, 8). Women saw greater gains in their share among faculty with less than three RPGs (from 5.3% in 1970 to 35.3% in 2022; dy/dx = 0.554, p = 0.000) than among super investigators (2.0% to 27.7%; dy/dx = 0.408, p = 0.000) (Fig 5A; S6-S7 Tables). These steeper gains among faculty with fewer RPGs translated to a lower RI for women among faculty super investigators across the period studied (0.2 in 1970; 0.6 in 2022) compared to the RI for women among NIH-funded faculty with less than three RPGs (0.4 in 1970; 0.8 in 2022) (Fig 5B). Correspondingly, the RI values for men with three or more RPGs declined less steeply (dy/dx = −0.408, p = 0.000; S7 Table) than for those with less than three RPGs (dy/dx = −0.554, p = 0.000; S6 Table), meaning that the gender gap remained wider for “super investigators” than for faculty with fewer RPGs, and that progress toward gender parity progressed more slowly among highly-funded faculty.

The share of white NIH-funded faculty declined across both funding levels, more steeply among super investigators (from 98% to 65%; slope = −0.608, p = 0.000) than investigators with fewer RPGs (95% to 68%; slope = −0.539, p = 0.000) (Fig 6A-6B; S6-S7 Tables). Between 1970 and 2022, Asian faculty increased from 3% to 24% among NIH-funded faculty with fewer than three RPGs (dy/dx = 0.399, p = 0.000; S6 Table), and from 2% to 26% among super investigators (dy/dx = 0.505, p = 0.000; S7 Table). Black, Hispanic, and other race faculty each made up less than 2% of faculty in both funding levels and saw no significant trends. Multiracial/ethnic faculty grew to represent about 5% of both subgroups of NIH-funded faculty by 2022 (<3 and 3 + RPGs: dy/dx = 0.09, p = 0.000; S6-S7 Tables).

RI trends by race/ethnicity further reveal persistent disparities (Fig 6C-D). White faculty remained overrepresented at both funding levels, with no significant change over time in either case. Among faculty with less than three RPGs, the RI for Hispanic faculty increased (dy/dx = 0.003, p = 0.001; S6 Table), while other race faculty saw a decline (dy/dx = −0.013, p = 0.000; S6 Table) and Black faculty saw no significant trend. No significant RI trends were observed for Black, Hispanic, or other race super investigators, whose RIs remained lower than they were among faculty with fewer RPGs (e.g., 0.2 vs. 0.4 among Black faculty), while the RI of multiracial/ethnic super investigators declined over time (dy/dx = −0.013, p = 0.000; S7 Table). Like the wider gender gap among super investigators, the racial/ethnic gaps between white faculty and faculty from underrepresented racial/ethnic groups remained wider among super investigators than those with fewer RPGs over time, indicating slower progress toward balanced racial/ethnic representation among highly funded faculty.

Finally, examining time trends stratified by NIH funding across intersectional gender and race/ethnicity groups offers a nuanced understanding of the patterns. In 1970, white men, white women, and Asian men accounted for nearly all NIH-funded faculty: 97.8% of those with less than three RPGs and 100% of super investigators (Fig 7A-D). By 2022, these three groups remained the majority of faculty PIs – 83.1% of faculty with less than three RPGs and 85.2% of those with more than three RPGs – but the distribution shifted: white men’s share fell to 44% (<3 RPGs) and 47% (3 + RPGs), while white women’s share increased to 24% and 19%, respectively, reflecting steeper gains among women faculty with less than three RPGs (dy/dx = 0.354, p = 0.000; S6 Table) than among white women faculty super investigators (dy/dx = 0.266, p = 0.000; S7 Table). In contrast, Asian men saw greater gains among super investigators (20% in 2022; slope = 0.401, p = 0.000; S7 Table) than among faculty with less than three RPGs (15% in 2022; slope = 0.260, p = 0.000; S6 Table).

By 2022, Asian women represented 8% of faculty with less than three RPGs and 6% of all faculty with 3 or more RPGs, reflecting significant increases in their representation within both funding levels (dy/dx = 0.139 and 0.104, respectively; p = 0.000; S6-S7 Tables). In contrast, Black and other race men and women and Hispanic women remained below 1% of both groups. Multiracial/ethnic women reached about 1.5% of both funding levels by 2022, reflecting slight increases over time within both groups (<3 RPG: dy/dx = 0.033, p = 0.000; 3 + RPG: dy/dx = 0.027, p = 0.015). Finally, multiracial/ethnic men saw slight increases in their representation over time (dy/dx = 0.06, p = 0.000 for both funding levels; S6-S7 Tables) and made up roughly 3% of each group by 2022.

RI trends reinforce these observed patterns (Fig 8A-8B). By 2022, white, Asian, and multiracial/ethnic men had RIs above 1 in each funding group. Asian men were especially overrepresented among super investigators compared to their relative representation among those with fewer RPGs (RI = 1.6 vs 1.2). In contrast, Black and Hispanic men remained underrepresented (RI < 0.7) with no significant RI trend over time among super investigators, and slight improvements in relative representation among less-funded investigators (dy/dx = 0.003, p = 0.000 for both).

Meanwhile, the RI for all racial/ethnic groups of women remained more closely clustered below 1 in both funding groups (Fig 8C-8D), although a growing gap widened in the past two decades. By 2022 the RI values for white and Asian women with less than three RPGs were 0.9 and 0.8, respectively, and 0.7 and 0.6, respectively, among faculty super investigators. Notably, white and Asian women’s RI values increased at a steeper rate among faculty super investigators (white women: dy/dx = 0.007, p = 0.034; Asian women: dy/dx = 0.013, p = 0.000; S7 Table) than among less-funded faculty (white women: dy/dx = 0.006, p = 0.000; Asian women: dy/dx = 0.010, p = 0.000; S6 Table). The RIs for Black, Hispanic, and other race women were the lowest of all gender-race/ethnicity groups, especially among super investigators. While Black and Hispanic women saw upward trends in their <3 RPG RIs over time (Black women: dy/dx = 0.003, p = 0.000; Hispanic women: dy/dx = 0.007, p = 0.000; S6 Table), other women did not, and the super investigator RIs did not increase significantly for any of the three groups of women in the 53 years observed.

## 4. Discussion

**Fig 9:**
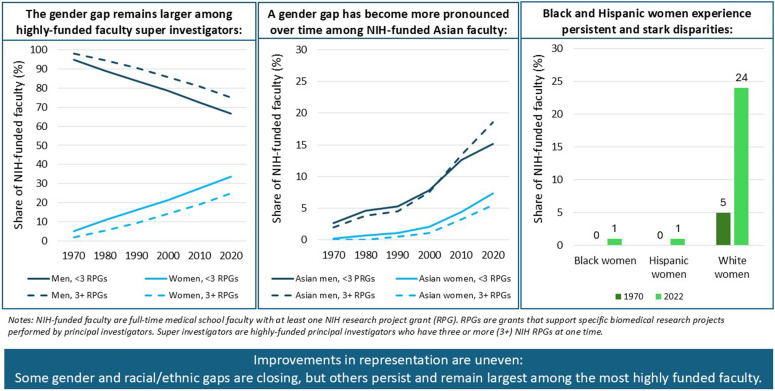
Demographic Trends among NIH-funded medical school faculty, 1970-2022: Key takeaways. (A) Trends in the gender distribution of NIH-funded faculty with <3 RPGs and 3 + RPGS. (B) Trends in the proportion of NIH-funded faculty with <3 RPGs and 3 + RPGs that were Asian men and Asian women. (C) Share of NIH-funded medical school faculty that were Black women, Hispanic women, and white women, 1970 vs. 2022.

Overall, this analysis of NIH-funded medical school faculty in the U.S. contributes two key insights to the existing literature on the research workforce within medical schools. First, it demonstrates that progress toward proportional representation among NIH-funded U.S. medical school faculty has lagged behind broader faculty diversity gains, highlighting persistent disparities in access to a critical resource for academic success: research funding [9]. For example, while medical school faculty have nearly reached balanced gender representation by 2022, women remain underrepresented among NIH-funded faculty relative to their representation among medical school faculty overall, and have seen slower progress toward gender equity among super investigators than less-funded faculty (Fig 9). Likewise, racial/ethnic groups who have historically been underrepresented within academic medicine (e.g., Black and Hispanic faculty) not only remained underrepresented within medical school faculty over time but also remained more starkly underrepresented among NIH-funded faculty *relative* to their representation among medical school faculty.

Second, these findings demonstrate the importance of intersectional analyses. As related studies have shown [10,15,26], disaggregating by gender and race/ethnicity reveal patterns that are otherwise obscured by aggregate statistics, like the overrepresentation of Asian men and persistent underrepresentation of Asian women among NIH-funded faculty (Fig 9). Without this level of detail, disparities may go unrecognized and, consequently, unaddressed. All together, these findings highlight mixed progress. Some groups, like white women and Asian men, have seen significant gains in representation, while others, including Hispanic and Black men – and most starkly, Hispanic and Black women – have seen little to no progress. These findings align with other studies, which have also identified a double bind experienced by women from underrepresented racial/ethnic groups [10,15,27]. There remains an urgent need for effective interventions to address these persistent inequities.

This study has some limitations. While this study focuses on NIH funding, this is not the only source of support for researchers at medical schools, so we do not yet have a clear picture of how race and gender are reflected in overall research funding. That said, the NIH remains the largest public source of biomedical research funding in the U.S. [28]. Second, the race/ethnicity variable is limited to broad categories which may conceal important within-group variation. For example, though Asian investigators are considered as a whole to be well-represented, some ethnic Asian groups are underrepresented in academic medicine. This limitation calls for more granular demographic data collection to allow for a more nuanced understanding of these trends and to better capture obscured disparities. The results for multiracial/ethnic and other race faculty also point to the importance of more granular racial and ethnic data. Without understanding the composition of these broad groups – for example, the extent to which the “multiracial/ethnic” category includes faculty with at least one underrepresented racial/ethnic identity versus faculty with multiple well-represented identities (e.g., white and Asian) – their results are less interpretable. Finally, the data used for this analysis are useful in documenting trends but are not well suited to examining explanations for the trends observed. For example, changes over time in faculty career tracks at medical schools [25] might explain some of the observed gender and racial/ethnic disparities in NIH funding, if there are demographic differences in the sub-populations of faculty on research tracks. Despite these limitations, these findings add new insight into demographic trends among a key population: NIH-funded medical school faculty.

The findings of this study also raise several questions for future research, particularly to better understand the mechanisms driving observed disparities. Future research could examine how findings may differ based on department type (e.g., clinical vs. basic science) or degrees held (e.g., MD/DO vs. PhD vs. MD/DO and PhD). Additionally, future analysis could move to individual-level analysis of patterns within faculty careers to explain some of these aggregated trends, such as whether women medical school faculty are experiencing higher rates of attrition from the NIH-funded workforce compared to men, and whether Black and Hispanic women are experiencing greater attrition from the NIH-funded faculty workforce than their white women peers. Prior analysis of funding longevity by gender found no significant gender gap [29], but we do not yet know if this finding differs within the academic medicine context or within certain racial/ethnic groups of women. Individual-level analysis can also help us better understand the structural barriers impeding increased representation of Black and Hispanic women, like gendered racism in research environments [30] and bias in grant review [8,9], as well as potential levers for change. Persistent disparities show that current efforts to increase diversity and the prevalence of Black and Hispanic women have not led to any noticeable gains or progress among NIH-funded medical school faculty. Future studies could examine the impact of investing in measures such as mentorship initiatives [30–32], additional grant initiatives targeting underrepresented groups [31], and material support to better support NIH-funded women and caregivers [31].

Although efforts exist to broaden the criteria for a successful research career, research grant funding remains key for establishing research independence, securing tenure, and being promoted. Persistent disparities in NIH funding represent disparities in the opportunity to reach these career milestones and remain in academic research, which may in turn perpetuate inequities in who chooses to pursue science [9].

## Supporting information

S1 Fig Temporal trends in the representation of racial/ethnic groups comprising less than 5% of NIH-funded full-time medical school faculty, 1970–2022. (TIF)

S2 Fig Temporal trends in the representation of gender-racial/ethnic groups comprising less than 5% of NIH-funded full-time medical school faculty, 1970–2022. (A) Trends in the representation of racial/ethnic groups of men comprising“ “less than 5% of NIH-funded medical school faculty.
(TIF)

S1 Table Temporal trends in the demographic distribution of full-time medical school faculty, by gender (Panel A), race/ethnicity (Panel B), and gender-race/ethnicity (Panel C). (XLSX)

S2 Table Temporal trends in the demographic distribution of NIH-funded full-time medical school faculty, by gender (Panel A), race/ethnicity (Panel B), and gender-race/ethnicity (Panel C). (XLSX)

S3 Table Temporal trends in the demographic distribution of NIH-funded full-time medical school faculty with <3 RPGs, by gender (Panel A), race/ethnicity (Panel B), and gender-race/ethnicity (Panel C). (XLSX)

S4 Table Temporal trends in the demographic distribution of NIH-funded full-time medical school faculty with 3 + RPGs, by gender (Panel A), race/ethnicity (Panel B), and gender-race/ethnicity (Panel C). (XLSX)

S5 Table Among All NIH-Funded Faculty, Slope of Proportion and Representation Index (RI) Over Time, 1970–2022. (PDF)

S6 Table Among NIH-Funded Faculty With < 3 RPGs, Slope of Proportion and Representation Index (RI) Over Time, 1970–2022. (PDF)

S7 Table Among NIH-Funded Faculty With 3 + RPGs, Slope of Proportion and Representation Index (RI) Over Time, 1970–2022. (PDF)
