# Supplementary figures and images for: Demographic trends and disparities among NIH-funded medical school faculty in the US, 1970–2022
Source: PLoS One. 2025 Dec 1;20(12):e0337610. doi: 10.1371/journal.pone.0337610 (PMC12668554; doi:10.1371/journal.pone.0337610)

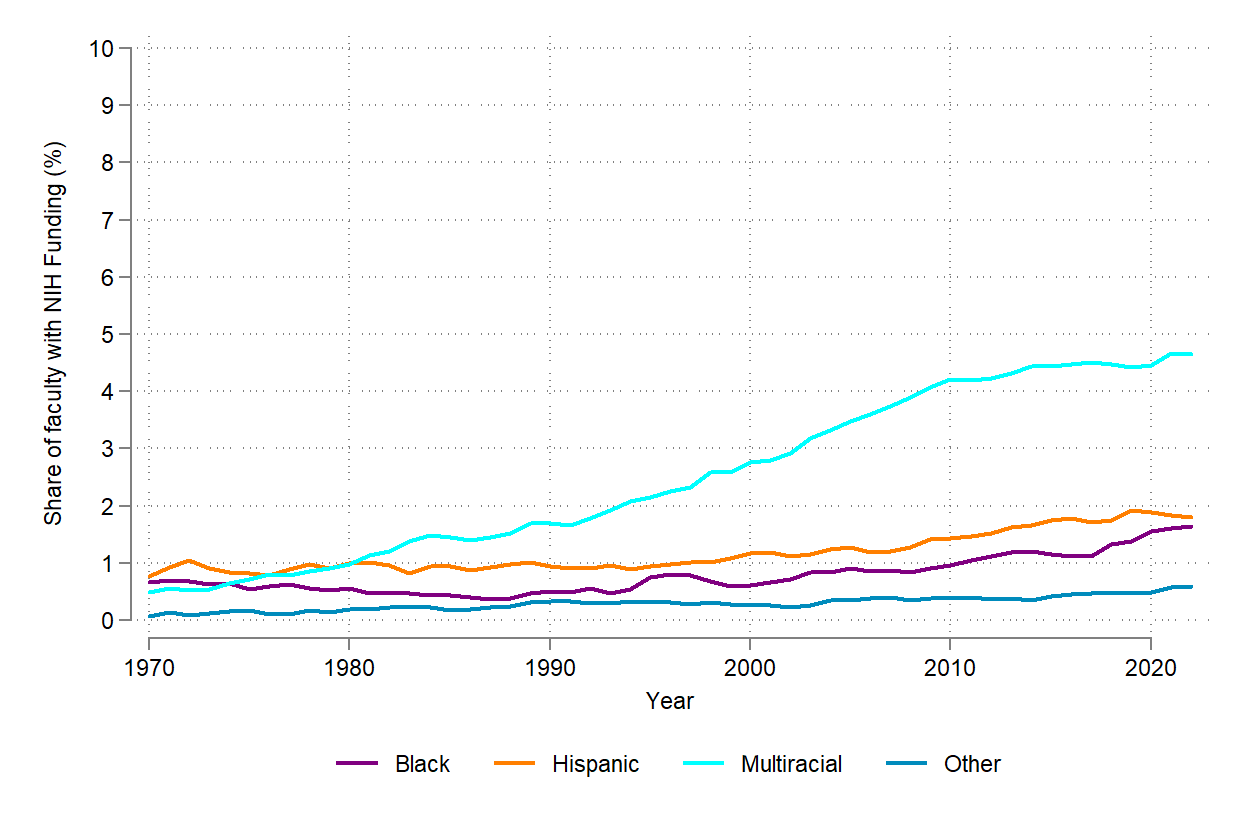

Supplement: S1 Fig — (TIF) [file pone.0337610.s001.tif]

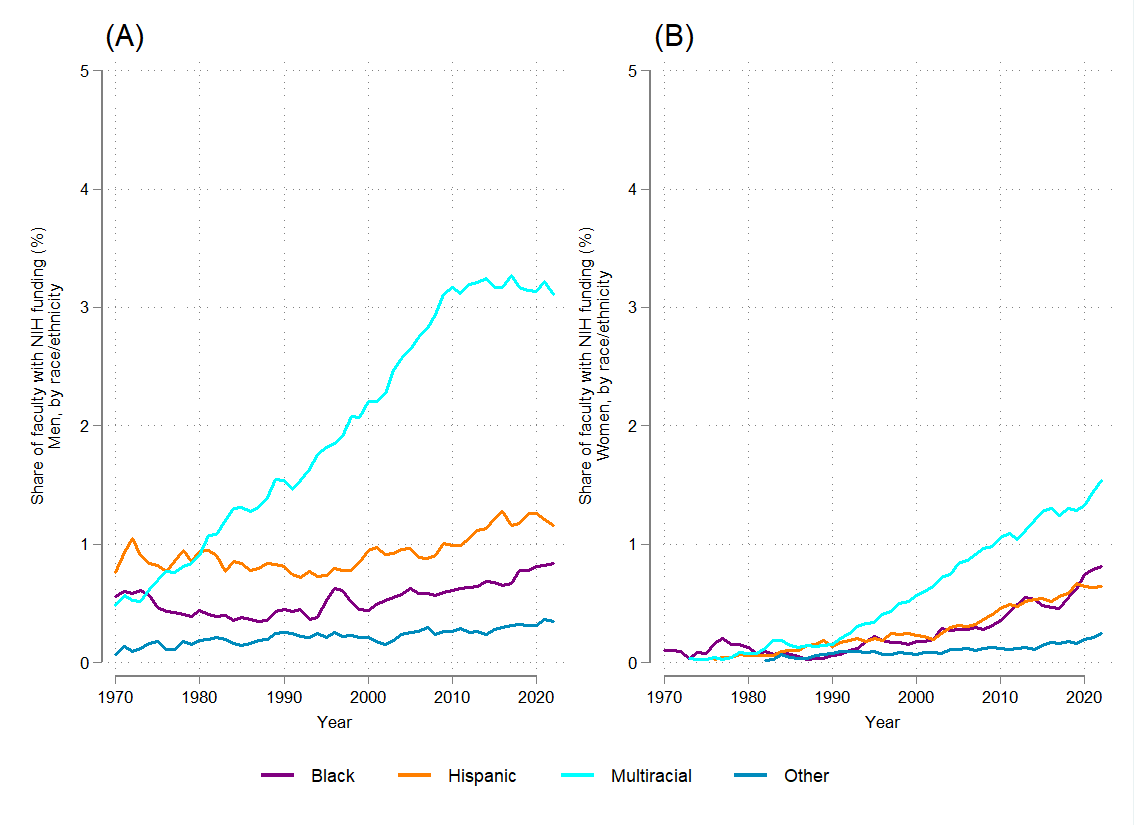

Supplement: S2 Fig — (A) Trends in the representation of racial/ethnic groups of men comprising“ “less than 5% of NIH-funded medical school faculty. (TIF) [file pone.0337610.s002.tif]
