# Supplementary material for: Demographic trends and disparities among NIH-funded medical school faculty in the US, 1970–2022
Source: PLoS One. 2025 Dec 1;20(12):e0337610. doi: 10.1371/journal.pone.0337610 (PMC12668554; doi:10.1371/journal.pone.0337610)
Supplement: S5 Table — (PDF) [file pone.0337610.s007.pdf]

S5 Table. Among All NIH-Funded Faculty, Slope of Proportion and Representation Index (RI) Over Time, 1970 to 2022

	Proportion		Representation Index (RI)	
	**Slope (dy/dx)**	**Statistically Significantly Different From:**	**Slope (dy/dx)**	**Statistically Significantly Different From:**
**Gender**				
Men (M)	-0.532***	W	0.002***	W
Women (W)	0.532***	M	0.005***	M
**Race/Ethnicity**				
White (Wh)	-0.537***	B, A, H, Mu, O	0.000	B, A, H, O
Black (B)	0.017	Wh, A, Mu	0.001	Wh, A, O
Asian (A)	0.401***	Wh, B, H, Mu, O	0.009***	Wh, B, H, Mu, O
Hispanic (H)	0.02	Wh, A, Mu	0.002***	Wh, A, O
Multiracial/ethnic (Mu)	0.092***	Wh, B, A, H, O	0.000	A, O
Other race/ethnicity (O)	0.007	Wh, A, Mu	-0.013***	Wh, B, A, H, Mu
**Gender-Race/Ethnicity**				
White men (WhM)	-0.876***	All	0.002**	WhW, AM, AW, HW, MuW, OM, OW
White women (WhW)	0.340***	All	0.006***	WhM, BM, BW, AM, AW, HM, MuM, OM, OW
Black man (BM)	0.006	WhM, WhW, AM, AW, MuM, MuW	0.003***	WhW, AM, AW, HW, MuW, OM, OW
Black woman (BW)	0.011	WhM, WhW, AM, AW, MuM, MuW	0.002**	WhW, AM, AW, HW, MuW, OM, OW
Asian man (AM)	0.267***	All	0.013***	All
Asian woman (AW)	0.134***	All	0.010***	All
Hispanic man (HM)	0.007	WhM, WhW, AM, AW, MuM, MuW	0.003***	WhW, AM, AW, HW, MuW, OM, OW
Hispanic woman (HW)	0.014	WhM, WhW, AM, AW, MuM, MuW	0.006***	WhM, BM, BW, AM, AW, HM, MuM, OM, OW
Multiracial man (MuM)	0.061***	All	0.003***	WhW, AM, AW, HW, MuW, OM, OW
Multiracial woman (MuW)	0.032***	All	0.007***	WhM, BM, BW, AM, AW, HM, MuM, OM, OW
Other race/ethnicity man (OM	0.004	WhM, WhW, AM, AW, MuM, MuW	-0.015***	All
Other race/ethnicity woman (OW)	0.004	WhM, WhW, AM, AW, MuM, MuW	-0.007***	All

*** p<0.01, ** p<0.05
