# Supplementary material for: Demographic trends and disparities among NIH-funded medical school faculty in the US, 1970–2022
Source: PLoS One. 2025 Dec 1;20(12):e0337610. doi: 10.1371/journal.pone.0337610 (PMC12668554; doi:10.1371/journal.pone.0337610)
Supplement: S6 Table — (PDF) [file pone.0337610.s008.pdf]

**S6 Table. Among NIH-Funded Faculty With < 3 RPGs, Slope of Proportion and Representation Index (RI) Over Time, 1970 to 2022**

| | **Proportion** | | **Representation Index (RI)** | |
|---|---|---|---|---|
| | **Slope (dy/dx)** | **Statistically Significantly Different From:** | **Slope (dy/dx)** | **Statistically Significantly Different From:** |
| **Gender** | | | | |
| Men (M) | -0.554*** | W | 0.002*** | W |
| Women (W) | 0.554*** | M | 0.006*** | M |
| | | | | |
| **Race/Ethnicity** | | | | |
| White (Wh) | -0.539*** | B, A, H, Mu, O | 0.000 | A, H, O |
| Black (B) | 0.019 | Wh, A, Mu | 0.001 | A, O |
| Asian (A) | 0.399*** | Wh, B, H, Mu, O | 0.009*** | Wh, B, H, Mu, O |
| Hispanic (H) | 0.021 | Wh, A, Mu | 0.003*** | Wh, A, O |
| Multiracial/ethnic (Mu) | 0.092*** | Wh, B, A, H, O | 0.001 | A, O |
| Other race/ethnicity (O) | 0.008 | Wh, A, Mu | -0.013*** | Wh, B, A, H, Mu |
| | | | | |
| **Gender-Race/Ethnicity** | | | | |
| White men (WhM) | -0.893*** | All | 0.002 | WhW, AM, AW, HW, MuW, OM, OW |
| White women (WhW) | 0.354*** | All | 0.006*** | WhM, BM, BW, AM, AW, HM, MuM, OM, OW |
| Black man (BM) | 0.006 | WhM, WhW, AM, AW, MuM, MuW | 0.003*** | WhW, AM, AW, HW, MuW, OM, OW |
| Black woman (BW) | 0.012** | WhM, WhW, AM, AW, MuM, MuW | 0.003*** | WhW, AM, AW, HW, MuW, OM, OW |
| Asian man (AM) | 0.260*** | All | 0.012*** | WhM, WhW, BM, BW, HM, HW, MuM, MuW, OM, OW |
| Asian woman (AW) | 0.139*** | All | 0.010*** | WhM, WhW, BM, BW, HM, MuM, MuW, OM, OW |
| Hispanic man (HM) | 0.007 | WhM, WhW, AM, AW, MuM, MuW | 0.003*** | WhW, AM, AW, HW, MuW, OM, OW |
| Hispanic woman (HW) | 0.015** | WhM, WhW, AM, AW, MuM | 0.007*** | WhM, BM, BW, AM, HM, MuM, OM, OW |
| Multiracial man (MuM) | 0.061*** | All | 0.003*** | WhW, AM, AW, HW, MuW, OM, OW |
| Multiracial woman (MuW) | 0.033*** | WhM, WhW, BM, BW, AM, AW, HM, MuM, OM, OW | 0.007*** | WhM, BM, BW, AM, AW, HM, MuM, OM, OW |
| Other race/ethnicity man (OM | 0.004 | WhM, WhW, AM, AW, MuM, MuW | -0.015*** | All |
| Other race/ethnicity woman (OW) | 0.004 | WhM, WhW, AM, AW, MuM, MuW | -0.007*** | All |

*** p<0.01, ** p<0.05
