# Supplementary material for: Demographic trends and disparities among NIH-funded medical school faculty in the US, 1970–2022
Source: PLoS One. 2025 Dec 1;20(12):e0337610. doi: 10.1371/journal.pone.0337610 (PMC12668554; doi:10.1371/journal.pone.0337610)
Supplement: S7 Table — (PDF) [file pone.0337610.s009.pdf]

S7 Table. Among NIH-Funded Faculty With 3+ RPGs, Slope of Proportion and Representation Index (RI) Over Time, 1970 to 2022

	Proportion		Representation Index (RI)	
	**Slope (dy/dx)**	**Statistically Significantly Different From:**	**Slope (dy/dx)**	**Statistically Significantly Different From:**
**Gender**				
Men (M)	-0.408***	W	0.005***	--
Women (W)	0.408***	M	0.007***	--
**Race/Ethnicity**				
White (Wh)	-0.608***	B, A, H, Mu, O	-0.001	A, Mu
Black (B)	0.012	Wh, A, Mu	0.002	A, Mu
Asian (A)	0.505***	Wh, B, H, Mu, O	0.019***	Wh, B, H, Mu, O
Hispanic (H)	0.002	Wh, A, Mu	-0.003	A, Mu
Multiracial/ethnic (Mu)	0.088***	Wh, B, A, H, O	-0.013***	Wh, B, A, H, O
Other race/ethnicity (O)	0.002	Wh, A, Mu	0.001	A, Mu
**Gender-Race/Ethnicity**				
White men (WhM)	-0.875***	All	0.003	AM, AW, MuM, MuW
White women (WhW)	0.266***	All	0.007**	AM, MuM
Black man (BM)	0.006	WhM, WhW, AM, AW, MuM	0.003	AM, AW, MuM, MuW
Black woman (BW)	0.006	WhM, WhW, AM, AW, MuM	0.003	AM, AW, MuM, MuW
Asian man (AM)	0.401***	All	0.027***	All
Asian woman (AW)	0.104***	All	0.013***	WhM, BM, BW, AM, HM, HW, MuM, OM
Hispanic man (HM)	-0.002	WhM, WhW, AM, AW, MuM	-0.002	AM, AW, MuW
Hispanic woman (HW)	0.005	WhM, WhW, AM, AW, MuM	0.001	AM, AW, MuM, MuW
Multiracial man (MuM)	0.063***	All	-0.011***	WhM, WhW, BM, BW, AM, AW, HW, MuW, OM, OW
Multiracial woman (MuW)	0.027**	WhM, WhW, AM, AW, MuM	0.014***	WhM, BM, BW, AM, HM, HW, MuM, OM
Other race/ethnicity man (OM	0.000	WhM, WhW, AM, AW, MuM	-0.001	AM, AW, MuM, MuW
Other race/ethnicity woman (OW)	0.002	WhM, WhW, AM, AW, MuM	0.005	AM, MuM

*** p<0.01, ** p<0.05
